# Interaction between NFATc2 and the transcription factor Sp1 in pancreatic carcinoma cells PaTu 8988t

**DOI:** 10.1186/s12867-017-0097-9

**Published:** 2017-08-03

**Authors:** Manuela Malsy, Bernhard Graf, Katrin Almstedt

**Affiliations:** 1 Department of Anesthesiology, University Medical Center Regensburg, Regensburg, Germany; 2 Department of Obstetrics and Gynecology, University Hospital Mainz, Mainz, Germany

**Keywords:** NFATc2, Sp1, Pancreatic carcinoma, Binding partner, Cancer

## Abstract

**Background:**

Nuclear factors of activated T-cells (NFATs) have been mainly characterized in the context of immune response regulation because, as transcription factors, they have the ability to induce gene transcription. NFAT proteins are found in several types of tumors, for instance, pancreatic carcinoma. The role of NFATs in carcinogenesis is regulating central genes in cell differentiation and cell growth. NFAT proteins are primarily located in cytoplasm and only transported to the cell nucleus after activation. Here, they interact with other transcription factors cooperating with NFAT proteins, thus influencing the selection and regulation of NFAT-controlled genes. To identify and characterize possible interaction partners of the transcription factor NFATc2 in pancreatic carcinoma cells PaTu 8988t.

**Methods:**

NFATc2 expression and the mode of action of Ionomycin in the pancreatic tumor cell lines PaTu 8988t were shown with Western blotting and immunofluorescence tests. Potential partner proteins were verified by means of immunoprecipitation and binding partners, their physical interactions with DNA pull-down assays, siRNA technologies, and GST pull-down assays. Functional evidence was complemented by reporter–promoter analyses.

**Results:**

NFATc2 and Sp1 are co-localized in cell nuclei and physically interact at the NFAT target sequence termed NFAT-responsive promotor construct. Sp1 increases the functional activity of its binding partner NFATc2. This interaction is facilitated by Ionomycin in the early stimulation phase (up to 60 min).

**Conclusions:**

Oncological therapy concepts are becoming more and more specific, aiming at the efficient modulation of specific signal and transcription pathways. The oncogenic transcription partner Sp1 is important for the transcriptional and functional activity of NFATc2 in pancreatic carcinoma. The binding partners interact in cells. Further studies are necessary to identify the underlying mechanisms and establish future therapeutic options for treating this aggressive type of tumor.

## Background

Malignant tumors are one of the worst scourges of humanity. Approximately 8.2 million people died of carcinoma in the year 2012 alone, a figure the World Health Organization estimates to rise to 13 million over the next 2 decades [1]. Pancreatic adenocarcinoma is one of the most aggressive types of malignant tumors in humans [2]. The main reasons for its unfavorable prognosis is the combination of rapid tumor growth, early-onset metastasis, and the availability of so far only inadequate diagnostic and therapeutic options [3]. The oncogenic potential of pancreatic tumor cells is triggered by the activation of oncogenic signal cascades and the altered regulation of important transcription factors [4]. Recent studies have indicated the importance of NFAT transcription factors in the carcinogenesis of pancreatic carcinoma [5].

The abbreviation NFAT stands for ‘nuclear factor of activated T-cells’. These factors are not only found in T-cells but also in other cells beyond the immune system, in which they regulate the expression of central genes with regard to cell differentiation and cell growth [6]. Additionally, NFAT proteins are also expressed in neural tissue, endothelial cells, the myocardial muscle, blood vessels, chondrocytes, keratinocytes, adipocytes, as well as in pancreatic tumor cells [7, 8].

NFAT proteins are primarily located in cytoplasm and only transported into the cell nucleus after activation. The calcium–calcineurin-NFAT signaling pathway is activated by an influx of calcium into the cell that leads to the dephosphorylation of NFAT, which allows it to enter the cell nucleus, thus increasing its DNA-binding affinity [9]. NFAT proteins are reverted to their original deactivated state in cytoplasm by rephosphorylation and nuclear export receptors [10].

Promoters of many genes that are highly important in regulating vital cell functions, such as cell proliferation, cell differentiation, survival, or programmed cell death, have binding sites for NFAT proteins, which—at least potentially—regulate these promoters at the expression level [11]. In fact, NFAT proteins may both stimulate and inhibit cell growth [12]. Such pluripotent effects depend on the type and activation status of the cell. In this respect, partner proteins seem to be of special significance [13]. Partner proteins are transcription factors cooperating with NFAT proteins and thus substantially influence the selection and regulation of NFAT-controlled genes [14]. The present work is focused on NFATc2 because of its high expression intensity in pancreatic carcinoma [15]. Yet, the function and DNA-regulating characteristics of NFATc2 are still unknown.

This study aimed at identifying and characterizing possible interaction partners of the transcription factor NFATc2 in pancreatic tumor cells.

## Methods

### Cell lines

The human pancreatic adenocarcinoma cell lines PaTu 8988t were obtained from H. P. Elsässer (Philipps University of Marburg, Germany) and maintained in Dulbecco’s modified Eagle’s medium (Sigma-Aldrich) supplemented with 10% fetal calf serum (FCS) (Sigma-Aldrich) and 1% Normocyn (Amaxa biosystems). Cells were cultured at 37 °C in humidified CO_2_ atmosphere (5%) and maintained in monolayer culture. Experiments were done with cells at ~70–80% confluence.

### Antibodies and reagents

Ionomycin was purchased from Sigma-Aldrich. For immunoblotting, membranes were probed with antibodies against NFATc2 (Santa cruz), Sp1 (Santa cruz), MEF 2A (Upstate cell signaling solutions), Lamin B (Santa cruz), and ß-actin (Sigma-Aldrich).

### Fluorescence microscopy

PaTu 8988t cells were grown on chambered coverslips, either left untreated or treated with 10% FCS, and subjected to transfection with the indicated plasmids (NFATc2-GFP). Cells were washed, fixed with 4% paraformaldehyde, blocked, and probed with NFATc2 (Santa cruz) and Sp1 (Santa cruz) antibodies. Proteins of interest were detected by means of fluorochrome-conjugated secondary antibodies (Invitrogene), and nuclei were counterstained with 4′6-diamino-2-phenylindole (DAPI). Cells were evaluated with a fluorescence microscope (Zeiss, Oberkochen, Germany).

### Subcellular fractionation, co-immunoprecipitation, and immunoblotting

For subcellular fractionation, cells were washed twice with cold DPBS and re-suspended in lysis buffer (12.5 mL 1 M HEPES, ph 7.5, 7.5 mL 5 M NaCl, 1.25 mL 200 mM EGTA, 25 mL 100% Gycerin, 2.5 mL Triton X-100, 1.05 g NaF, 1.11 g Na_4_P_2_O_7_ × 10 H_2_O) containing the protease inhibitors Orthovandat (Sigma aldrich), Leupeptin (Sigma aldrich), Benzamidin (Sigma aldrich), PMSF (Sigma aldrich), Aprotinin (Sigma aldrich). After sonification, cells were centrifuged at 13.000 rpm for 5 min, and supernatants were transferred to new cups and incubated on ice.

For co-immunoprecipitation, 500 μg of the lysates was immunoprecipitated with 4 µL of the indicated antibodies and protein G or A agarose (Roche Diagnostics). The immunoprecipitates were subjected to immunoblotting.

For Western blotting, protein extracts were analyzed by SDS–PAGE and blotted onto nitrocellulose. Upon protein extraction and gel transfer, membranes were washed in TBS washing buffer and incubated with peroxidase-conjugated secondary antibodies. Immunoreactive proteins were visualized by means of an enhanced chemiluminescence detection system (Western blotting detection reagent, GE healthcare). Membranes were probed with anti-NFATc2 (Santa Cruz), anti-Sp1 (Santa cruz) and anti-MEF 2A (Upstate cell signaling solutions). Anti-Lamin B (Santa cruz) and anti-ß-actin (Sigma-Aldrich) antibodies were used as loading control.

### DNA pull-down assay

For DNA pull-down assay, 200 μg proteins per sample was incubated for 3 h with 10 μL of biotinylated double-stranded oligonucleotides containing the GGAAA consensus NFAT binding sequence of the human interleukin-2 promoter (5′-tctaaggaggaaaaactgtttcatg-3′ and its complementary strand) (Biomers.net GmbH). DNA–protein complexes were further incubated with 60 µL streptavidin-agarose beads (Sigma-Aldrich) for 1 h, washed twice with lysis buffer, and subjected to immunoblotting.

### Preparation of the recombinant GST-NFAT proteins and GST pull-down assay

Bacteriologically expressed GST fusion proteins were coded by pGEX plasmids. We used BL21 strains of *Escherichia coli* for amplification of the pGEX GST-NFAT plasmid and protein extraction. The transformed colonies were inoculated with 5 mL of LB medium (Roth) and 5 µL of ampicillin (Sigma-Aldrich), and the culture was incubated at 37 °C on an orbital shaker for 12–15 h (up to OD660 of 0.2–2.0). Expression of NFAT fusion proteins was induced by adding 0.75 mL of IPTG solution (AppliChem). Bacteria were lysed by sonification, and we identified the produced proteins by means of SDS–polyacrylamide gel electrophoresis. For the actual assay, we incubated 100 µL of purified glutathione agarose beads (GE Healthcare) with 3 µg of bacteriologically expressed GST or GST-NFAT and total protein at 4 °C for 15–18 h. After centrifugation and several wash cycles, samples were mixed with 30 µL of Laemmli puffer, heated up to 95 °C for 5 min, and analyzed by Western blotting.

### Transient transfection, siRNA, and luciferase reporter assay

Cells were seeded in 12-well plates. For transient transfection of expression constructs, PaTu 8988t cells were transfected 24 h after seeding at 70% density using TransFast (Promega) as a transfection reagent according to the manufacturer’s instructions. The promoter constructs cisNFAT-Luc were kindly provided by Stratagene Garden Grove, USA.

Luciferase activity was measured using the Lumat LB 9501 (Berthold Technologies, Mannheim, Germany) luminometer and the dual Luciferase Reporter Assay System (Promega) according to the manufacturer’s instructions. Firefly luciferase values were normalized to Renilla luciferase activity and are shown as mean values ± SD.

For siRNA transfection, we obtained NFATc2 siRNA (5′-CCAUUAAACAGGAGCAGAAtt-3′), Sp1 siRNA (5′-GGUAGCUCUAAGUUUUGAUtt-3′), and the Silencer Negative Control from Ambion (applied biosystems). Cells were transfected for 24 h using the siLentFect lipid reagent (Biorad) according to the manufacturer’s protocol.

## Results

### NFATc2 becomes translocated into the cell nucleus in the presence of Ionomycin

**Fig. 1:**
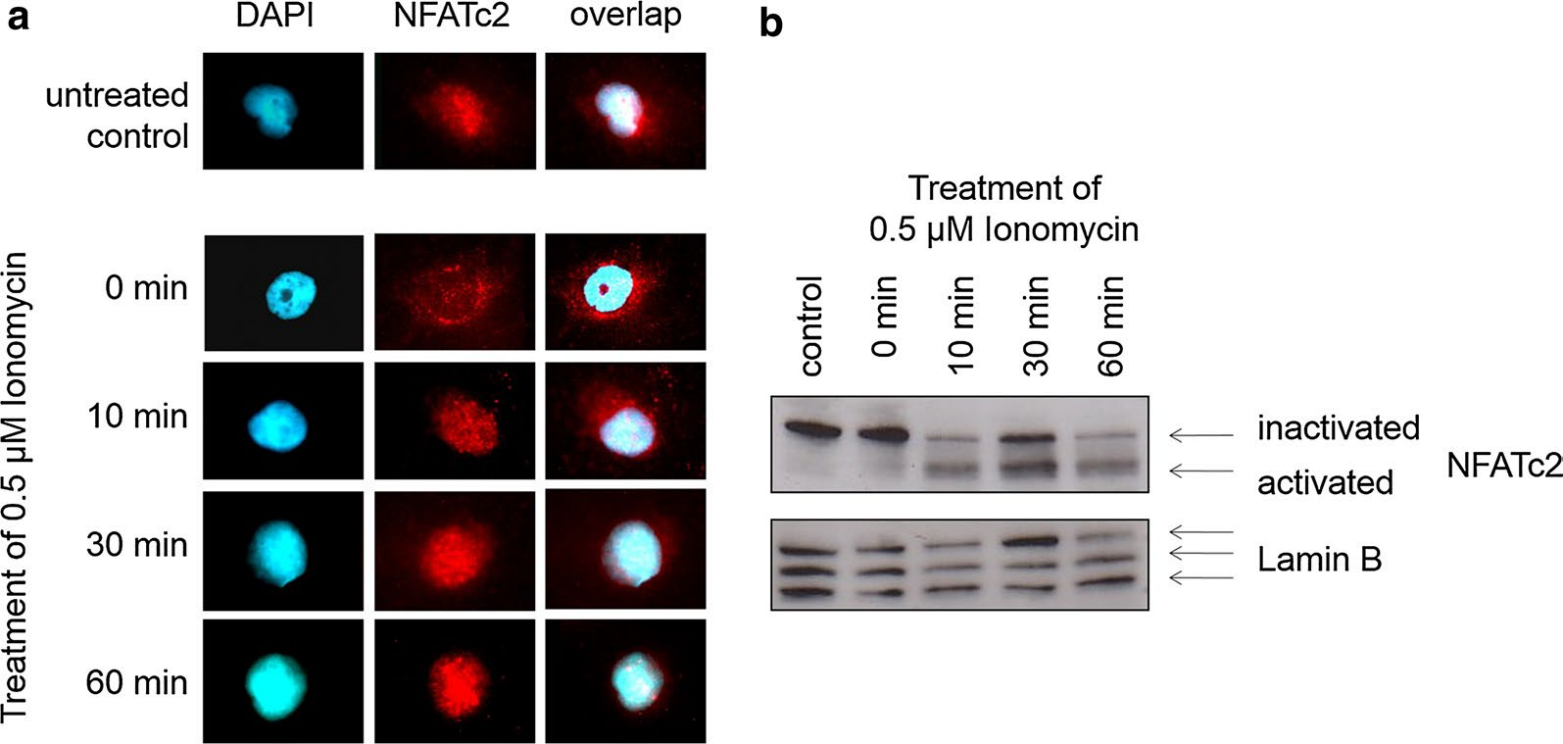
NFATc2 becomes translocated into the cell nucleus after stimulation with Ionomycin. Pancreatic tumor cells mixed with serum-free medium for 3 h were stimulated with 0.5 µM of Ionomycin for 0, 10, 30, and 60 min. Evidence is provided by means of NFATc2 antibodies in Western blot analysis (**b**) or by immunofluorescence (**a**). Cell nuclei (*blue*) are stained with DAPI and endogenous NFATc2 (*red*) with alexarot

Interaction between NFATc2 and potential partner proteins in regulating transcription necessitates the reliable translocation of NFATc2 into the cell nucleus with the aid of a stimulant. Ionomycin is the stimulant of choice because influx of calcium into the cell activates the calcium–calcineurin-NFAT signaling pathway that leads to the dephosphorylation of NFAT, allowing it to enter the cell nucleus, and thus increases its DNA-binding affinity. Immunofluorescent images of untreated cells (Fig. 1a) showed the presence of NFATc2 in the entire cell. In contrast, when a serum-free medium was added, NFATc2 was only present in cytoplasm. After 10-min stimulation with Ionomycin, NFATc2 was translocated into the cell nucleus. This translocation had its maximum at 30 min and was still present after 60 min. The overlap of nucleus staining with DAPI confirmed the location of NFATc2 in the cell nucleus after stimulation with Ionomycin. This translocation by Ionomycin could also be proven at the protein level by means of Western blotting (Fig. 1b).

### Endogenous expression of NFATc2 partner proteins described in the literature and their physical interaction during immunoprecipitation

We identified already established partner proteins of NFAT in PaTu 8988t pancreatic carcinoma cells. These partner proteins have already been described in the literature for other organ systems and additionally fulfill the following criteria:

1. calcium-dependent regulation, and
2. verified oncogenic effect in the organism.

The effect was proven at the protein level by means of Western blot analysis. Subsequently, we investigated protein–protein interactions in vivo by means of immunoprecipitation.

**Fig. 2:**
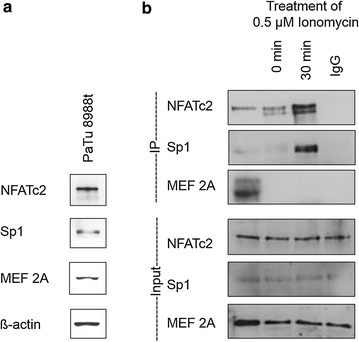
Endogenous expression of NFATc2 partner proteins described in the literature and their physical interaction during immunoprecipitation. Proteins were verified in Western blot analysis using the antibodies NFATc2, Sp1, and MEF 2A as well as the loading control ß-actin (**a**). NFATc2 including its interaction partner was subsequently extracted by means of immunoprecipitation (**b**)

Figure 2a shows the strong expression of both NFATc2 and its potential partner proteins Sp1 and MEF 2A. Row 1 of Fig. 2b shows immunoprecipitation of NFATc2 in relation to the duration of the stimulation with Ionomycin. In contrast to serum-free cells that only precipitated little NFATc2, stimulation with Ionomycin yielded a significantly more intense signal. When verifying the potential partner proteins Sp1 and MEF 2A (row 2 and 3) expressed in pancreatic carcinoma cells, Sp1 could also be precipitated as a binding partner of NFATc2. The signal strength of Sp1 also significantly increased during stimulation with Ionomycin. MEF 2A could not be proven as a potential binding partner. Row 4–6 show the input controls.

### NFATc2 co-localizes with Sp1 in the same immunocomplex

**Fig. 3:**
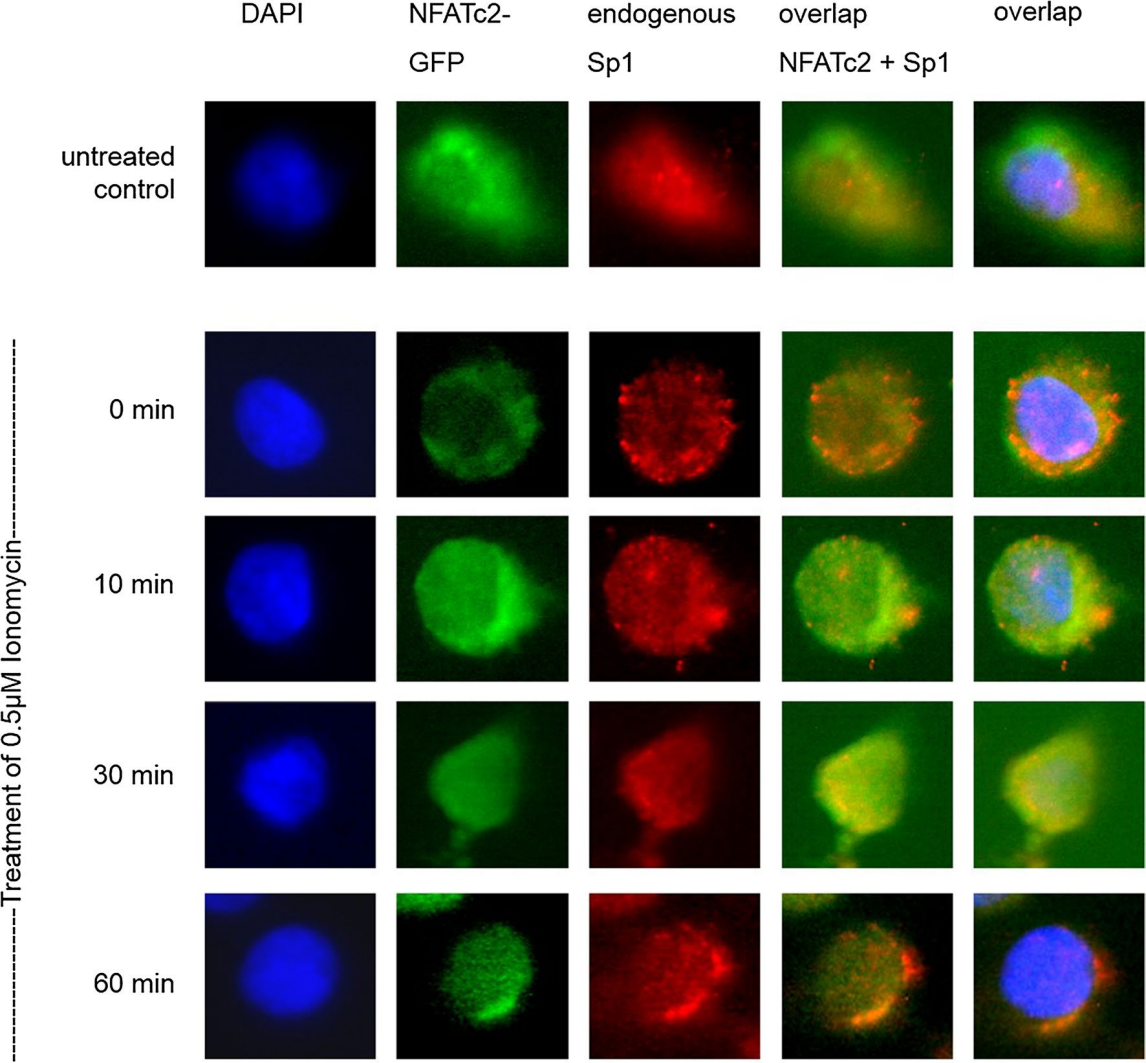
Co-localization of NFATc2 and the transcription factor Sp1. PaTu 8988t cells were transiently transfected with NFATc2-GFP. The cells either remained in serum containing medium or, after 24 h, serum was removed for 3 h, and the cells were stimulated with 0.5 µM of Ionomycin for 0, 10, 30, or 60 min. The cells were fixed into paraformaldehyde and incubated with the Sp1 antibody. Cell nuclei (*blue*) are stained with DAPI, endogenous Sp1 (*red*) with alexarot, and NFATc2-GFP *green*

The possible co-localization of NFATc2 and Sp1 in cell nuclei was investigated by means of immunofluorescence (Fig. 3). In untreated cells, NFATc2-GFP was present in the entire cell. In contrast, when a serum-free medium was added, NFATc2 was only present in cytoplasm. After stimulation with Ionomycin for 10 and 30 min, NFATc2 was translocated into the cell nucleus. Translocation was at its maximum after 60 min. Sp1 did not seem to be subject to any regulation by Ionomycin and was mainly observed in the cell nucleus.

The overlap of NFATc2-GFP with Sp1 indicated Ionomycin-induced co-localization of the two partner proteins in cell nuclei that could be verified by the additional overlap of nucleus staining with DAPI.

### Co-immunoprecipitation of NFATc2 and Sp1 complexes of pancreatic tumor cells

**Fig. 4:**
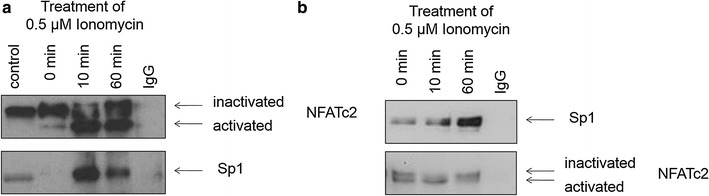
Co-immunoprecipitation of NFATc2 and Sp1 complexes of pancreatic tumor cells. After 3 h incubation with serum-free medium, the cells were stimulated with 0.5 µM of Ionomycin, harvested, and lysed at defined time points (0, 10, and 60 min). NFATc2 or Sp1 proteins were immunoprecipitated by means of an antibody coupled to an agarose bead. Sp1 binding to NFATc2 (**a**) or NFATc2 binding to Sp1 (**b**) were analyzed by means of Western blotting

Supplementary immunoprecipitation experiments were conducted to examine the physical interaction between NFATc2 and Sp1 induced by Ionomycin with regard to the time interval. Using an antibody against NFATc2, two bands could be detected reflecting the different phosphorylation stages of the protein (Fig. 4a). 10-min stimulation with Ionomycin led to significant signal amplification of the activated dephosphorylized NFATc2 protein that was still present after 60 min stimulation. The lower bands reflect binding Sp1. Similar to NFATc2, 10 min stimulation with Ionomycin led to significant signal amplification of Sp1 protein that decreased after 1 h.

As conclusion, Ionomycin may facilitate the interaction between NFATc2 and Sp1 at the early stage of stimulation.

Sp1 was immunoprecipitated in an analogous manner. Co-immunoprecipitated NFATc2 was verified by means of Western blot analysis (Fig. 4b). Stimulation with Ionomycin significantly increased precipitation of Sp1. Co-immunoprecipitated NFATc2 was also found at each of the three time points of stimulation. After binding, inactive NFATc2 (time point 0 min) changed to activated NFATc2 after 10 min, whereas only few active NFATc2 proteins seemed to be binding to Sp1 after 60 min.

### NFATc2 and Sp1 physically interact at the NFAT target sequence

**Fig. 5:**
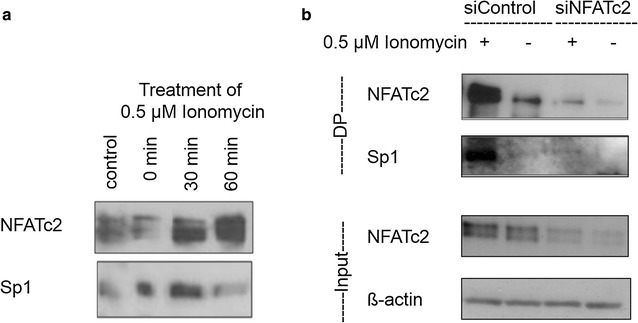
Physical interaction at the NFAT target sequence. DNA oligonucleotide, which includes the NFAT consensus binding sequence GGAAA, was either incubated with the cell lysate of cells stimulated with Ionomycin or untreated cells (**a**) or transiently transfected with ‘Silencer Negative Control’—siRNA or NFATc2-specific siRNA oligonucleotide and also stimulated or not stimulated (**b**). Proteins binding to the biotin-marked oligonucleotide sequence were subsequently precipitated with streptavidin. By means of the respective antibodies, NFATc2 and Sp1 were determined by Western blot analysis

Physical interactions between NFATc2 and Sp1 as well as their activity status in the DNA were identified by means of DNA pull-down assays (Fig. 5a). The pancreatic tumor cell lines PaTu 8988t were stimulated with Ionomycin for a certain period of time. We also examined the binding of NFATc2 and Sp1 to the oligonucleotide sequence containing the NFAT consensus binding sequence GGAAA. Stimulation with Ionomycin increased the DNA-binding affinity of NFATc2 that resulted in stronger NFATc2 binding to the NFAT target sequence. The fact that Sp1 could be co-precipitated as a physical interaction partner of NFATc2 indicates the joint binding of both proteins at the NFAT consensus sequence.

Apart from the 30 min stimulation with Ionomycin, endogenous expression of NFATc2 was inhibited by means of siRNA technology (Fig. 5b). We investigated the extent to which DNA interaction of the partner proteins could be prevented.

In analogy to Fig. 5a and the previous immunoprecipitation investigations (Fig. 4a, b), Fig. 5b shows that stimulation with Ionomycin increased promoter binding of NFATc2 and Sp1. Additional cell treatment with siRNA against NFATc2 decreased NFATc2-Sp1-DNA binding compared to siControl (row 1 and 2). The total cell lysate of the treated cells was directly applied to row 3 in the Western blot analysis to confirm the functionality of siRNA. Row 4 shows the endogenous expression of ß-actin as loading control.

### NFATc2 and Sp1 directly interact in vitro in GST pull-down assay

**Fig. 6:**
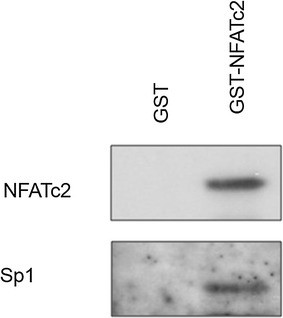
Direct interaction between NFATc2 and Sp1 in GST pull-down assay. Bacteriologically expressed GST and GST-NFATc2 as well as Sp1 previously over-expressed in the pancreatic tumor cells were examined by means of GST pull-down assay; precipitates were determined by Western blot analysis

GST pull-down assays were used to answer the question if the transcription factors Sp1 and NFATc2 physically interact in pancreatic tumor cells. We examined the binding of bacteriologically expressed GST-NFATc2 to Sp1 that had been overexpressed in the pancreatic carcinoma cell lines PaTu 8988t by transfection. Bacteriologically expressed GST fusion proteins that did not physically interact with the transcription partners were used as control. As a direct physical interaction partner, Sp1 could be precipitated with GST-NFATc2 fusion proteins by means of Western blot analysis (Fig. 6).

### Functional interaction between NFATc2 and Sp1

**Fig. 7:**
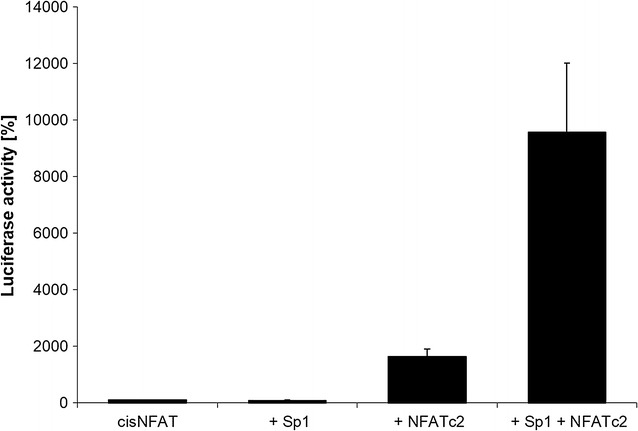
Functional interaction between NFATc2 and Sp1 in luciferase assay. The artificial NFAT-responsive reporter-promotor construct cisNFAT-Luc and the effector plasmids NFATc2 or Sp1, or both, were transiently transfected into the pancreatic tumor cell lines PaTu 8988t, and relative luciferase activity was determined. In the evaluation, the basal activity of the promoter construct cisNFAT-Luc is normalized to the value 100 and the influences of the regulatory changes are indicated in x-fold increase or reduction of this control. The test repeated three times

The functional relevance of NFATc2 and Sp1 binding to promoters was investigated with the aid of reporter-promoter analyses (Fig. 7). Here, we also transiently transfected the artificial NFAT-responsive reporter-promoter construct cisNFAT-Luc, which exclusively shows three consecutive activated NFAT binding sites, with the effector plasmids NFATc2 or Sp1 into the pancreatic tumor cells. For analysis, the basal activity of the promoter construct cisNFAT-Luc was normalized to the value of 100 followed by evaluation of the influences of regulatory changes. Transfection of the cisNFAT-Luc promoters showed the basal activity of the cell (column 1). Equally weak luciferase assay values were found after the co–transfection of Sp1 (column 2). Transfection of NFATc2 resulted in a 16-fold increase in luciferase activity in comparison to the basal expression of the promoter (column 3). Co–transfection of the transcription factors NFATc2 and Sp1 into the cell yielded a further sixfold activation of the reporter construct in comparison to NFATc2 activity alone (column 4). Thus, Sp1 may significantly increase the functional activity of NFATc2 binding to the promoter.

## Discussion

Binding partners are crucial for the specificity of transcription factors in cells. In recent years, numerous studies have shown that nuclear factors of activated T-cells (NFATs) are not only present in T-cells but also in cells beyond the immune system. As transcription factors, T–cells play an important role in inducing gene transfer [16], regulating and controlling numerous genes responsible for cell proliferation, cell differentiation, survival, and apoptosis [6]. This way, NFAT proteins influence central effects in tumor biology, such as stimulating angiogenesis by upregulation of VEGF [17], triggering the proliferation of tumor cells by upregulating c-myc [9], or promoting the migration of tumor cells by COX-2 [18].

The fact that all pluripotent effects depend on the type of cell and its activity status is remarkable. Partner proteins seem to be of particular importance in gene expression induced by NFATs [19]. Such proteins are transcription factors that cooperate with NFAT proteins, thus influencing the selection and regulation of NFAT-controlled genes [14]. However, little is yet known about these binding partners.

The probably most well-known binding partner of NFAT is the transcription factor AP-1 (activator protein 1) in lymphocytes [20, 21]. But also FOXP3, MEF 2A, IRF4, and GATA have been identified. Together with NFAT, these transcription factors activate or repress important target genes necessary for T cell activation [14]. Several other transcription factors have been described in the context of other cell systems, for instance, EGR in neurons, CREB in the differentiation of osteoclasts [22], CCAAT/enhancer binding proteins (C/EBP) in hepatocytes [23], and Sp1 in keratinocytes [24]. Kao et al. described the interaction of Sox10 with NFATc3 in glial cells that ultimately contribute to myelination by regulating the Krox20 protein [25].

Little evidence exists on the effect partner proteins of NFATc2 have on the oncogenic behavior of pancreatic tumor cells. Therefore, the current work is focused on characterizing the binding partners of NFATc2 in pancreatic tumor cells and their verification by different molecular biological methods. The stimulant of choice was Ionomycin to guarantee the reliable translocation of NFATc2 into the cell nucleus. Stimulation with Ionomycin causes influx of calcium into the cell, activating the calcium–calcineurin-NFAT signaling pathway. This activation leads to the dephosphorylation of NFAT, which allows it to enter the cell nucleus, thus increasing its DNA-binding affinity [26]. The disadvantage of the stimulus is its cell toxicity that rules out long-term experiments. The mode of action of Ionomycin was proven by immunofluorescence as well as Western blot analysis.

First interaction experiments by means of immunoprecipitation showed binding of NFATc2 to the transcription factor Sp1. The family of Sp (specificity protein) transcription factors belongs to the zinc finger proteins and is divided into two sub-groups: the Sp-like factors Sp1 to Sp8 and the KLF-like factors KLF1 to KLF16 [27].

Sp-like transcription factors are highly important in eukaryotic transcription processes. By regulating the expression of multiple genes, these factors are involved in many cellular functions, such as cell proliferation, apoptosis, and differentiation, as well as in neoplastic transformation [28, 29]. Each family member differs in its ability to control transcription and regulate cellular processes [30]. Depending on the promoter and binding partner, Sp-like transcription factors may either have an activating or an inhibiting effect. KLF13, for instance, activates the promoters of the Simian virus (SV 40) and of γ-Globin but inhibits the cytochrome P450 CYP1A1 [31].

Even if the specific physiological function of Sp proteins is not yet fully understood, knock-out studies on mice have shown the involvement of this family of transcription factors in the development of tissue and organs. Sp protein expression is also assumed to be a critical factor in tumor development and growth as well as in metastasis [32]. Kumar and colleagues described increased expression and activity of Sp1 in epithelial carcinoma in comparison to benign tumors [33]. In 2001, Shi et al. found over-expression of Sp1 in pancreatic tumor cells compared to normal tissue [34]. Sp1 has also been identified as a mediator of TGF ß-induced tumor progression in pancreatic carcinoma [35].

The application of further techniques has shown the direct interaction of transcription factors in the same immunocomplex at the NFAT target sequence GGAAA. Sp1 increases the functional activity of NFATc2 at the NFAT-responsive promoter construct. Our immunofluorescent investigations showed that, in pancreatic tumor cells, NFATc2 stimulated with Ionomycin becomes translocated into the cell nucleus, co-locating with the oncogenic transcription factor Sp1.

Stimulating pancreatic tumor cells with Ionomycin seems to promote binding of the two partner proteins NFATc2 and Sp1. Immunoprecipitation studies have shown that mainly the respective partner is precipitated under stimulation. Notable are the different stages of phosphorylation of NFATc2. Activated dephosphorylized NFATc2 is able to increasingly bind Sp1. Vice versa, immunoprecipitation of Sp1 may lead to the co-precipitation of both activated and deactivated dephosphorylized NFATc2. One possible explanation may be that the binding partners NFATc2 and Sp1 interact, complex, and jointly regulate target genes in the DNA. Glycogen synthase kinase-3 (GSK-3) or casein kinase 1 (CK1) limit this process in time by re-phosporylizing NFAT [10], thereby causing the NFATc2-Sp1 complex to lose its DNA-binding affinity.

It remains unclear, however, to what extent Sp1 as a binding partner of NFATc2 has a direct influence on the transcriptional and functional activity of DNA in pancreatic carcinoma. To answer this question, we conducted DNA pull-down assays and luciferase assays. Our investigations showed that stimulation with Ionomycin increases DNA-binding of NFATc2 and co-precipitated Sp1. Furthermore, Sp1 increased the transactivation of the NFAT-responsive promoter construct.

The oncogenic transcription factor Sp1 plays an important role in the transcriptional and functional activity of NFATc2 in pancreatic carcinoma, in which the binding partners interact in the cell.

## Conclusions

Modern treatment strategies of tumor diseases are directed at the efficient modulation of specific signaling and transcription pathways. In this context, VEGF antibodies [36], tyrosine kinase inhibitors [37], or EGFR antibodies have been discussed for the treatment of advanced pancreatic carcinoma [38]. Fundamental to identifying new therapeutic approaches is comprehensive knowledge about the carcinogenesis [39]. The carcinogenic process involves many proteins that may act as transcription factors or co-factors, or both, with significant influence on the regulation of target genes. Next to the transcription factors NFATc2 and Sp1, many other proteins interactively influence transcription processes by regulating promoter activity as required and by controlling cell functions. Here, NFATc2 seems to have a key role in the progression of pancreatic carcinoma [5].

Many further studies are necessary to identify the underlying mechanisms. Identifying and characterizing central partner proteins in the carcinogenesis of pancreatic carcinoma will help establish new therapeutic options in the treatment of this aggressive type of tumor.

## Abbreviations

AP-1: activator protein-1
C/EBP: CCAAT/enhancer protein
CK1: casein kinase 1
COX-2: cyclooxygenase-2
CREB: cAMP response element binding protein
DAPI: 4′6-diamino-2-phenylindole
EGFR: epidermal growth factor receptor
EGR: early growth response gene
FCS: fetal calf serum
FOXP3: forkhead protein 3
GATA: GATA binding facctor
GSK 3: gycogensynthase kinase 3
KLF: kruppel-like transkriptionsfactor
MEF 2A: myocyte enhancer Factor 2A
NFAT: nuclear factors of activated T-cells
siRNA: small interfering RNA
Sp1: specificity protein 1
SV: 40 Simian virus 40
SOX10: SRY-Box 10
TGF ß: transforming growth factor beta
VEGF: vascular epithelial growth factor
